# Automated Diagnosis of Cervical Intraepithelial Neoplasia in Histology Images via Deep Learning

**DOI:** 10.3390/diagnostics12020548

**Published:** 2022-02-21

**Authors:** Bum-Joo Cho, Jeong-Won Kim, Jungkap Park, Gui-Young Kwon, Mineui Hong, Si-Hyong Jang, Heejin Bang, Gilhyang Kim, Sung-Taek Park

**Affiliations:** 1 Medical Artificial Intelligence Center, Hallym University Medical Center, Anyang 14068, Korea; jungkap.park@gmail.com; 2 Department of Ophthalmology, Hallym University Sacred Heart Hospital, Hallym University College of Medicine, Anyang 14068, Korea; 3 Department of Pathology, Kangnam Sacred Heart Hospital, Hallym University College of Medicine, Seoul 07441, Korea; uteotw@hallym.or.kr; 4 Seoul Clinical Laboratories, Yongin 16954, Korea; kw9202@scllab.co.kr; 5 Department of Pathology, Chung-Ang University Hospital, Chung-Ang University College of Medicine, Seoul 06973, Korea; hongmineui@gmail.com; 6 Department of Pathology, Soonchunhyang University Cheonan Hospital, Soonchunhyang University College of Medicine, Cheonan 31151, Korea; slogic001@gmail.com; 7 Department of Pathology, Konkuk University Medical Center, Konkuk University School of Medicine, Seoul 05030, Korea; mars-21@hanmail.net; 8 Department of Obstetrics and Gynecology, Kangnam Sacred Heart Hospital, Hallym University College of Medicine, Seoul 07441, Korea; parkst96@hallym.or.kr

**Keywords:** cervical intraepithelial neoplasia, histology image, artificial intelligence, deep learning, convolutional neural network

## Abstract

Artificial intelligence has enabled the automated diagnosis of several cancer types. We aimed to develop and validate deep learning models that automatically classify cervical intraepithelial neoplasia (CIN) based on histological images. Microscopic images of CIN3, CIN2, CIN1, and non-neoplasm were obtained. The performances of two pre-trained convolutional neural network (CNN) models adopting DenseNet-161 and EfficientNet-B7 architectures were evaluated and compared with those of pathologists. The dataset comprised 1106 images from 588 patients; images of 10% of patients were included in the test dataset. The mean accuracies for the four-class classification were 88.5% (95% confidence interval [CI], 86.3–90.6%) by DenseNet-161 and 89.5% (95% CI, 83.3–95.7%) by EfficientNet-B7, which were similar to human performance (93.2% and 89.7%). The mean per-class area under the receiver operating characteristic curve values by EfficientNet-B7 were 0.996, 0.990, 0.971, and 0.956 in the non-neoplasm, CIN3, CIN1, and CIN2 groups, respectively. The class activation map detected the diagnostic area for CIN lesions. In the three-class classification of CIN2 and CIN3 as one group, the mean accuracies of DenseNet-161 and EfficientNet-B7 increased to 91.4% (95% CI, 88.8–94.0%), and 92.6% (95% CI, 90.4–94.9%), respectively. CNN-based deep learning is a promising tool for diagnosing CIN lesions on digital histological images.

## 1. Introduction

In 2018, cervical cancer ranked as the fourth most frequently diagnosed cancer and the fourth leading cause of cancer-related death in women worldwide [1]. Despite the decreasing incidence in developed countries due to active screening and vaccination for human papilloma virus (HPV), its prevalence and mortality are increasing in sub-Saharan Africa, southeastern Asia, eastern Europe, and South America. Histologically, the most common type is squamous cell carcinoma, and HPV is the virtually necessary (but not sufficient) cause of cervical cancer [1]. For early detection, screening methods, such as the HPV test, cervical cytology, and colposcopy, are recommended. However, the gold standard for diagnosing cervical lesions is the microscopic evaluation of histopathology by a qualified pathologist [2].

Premalignant lesions of the cervix, cervical intraepithelial lesions (CINs) are proliferations of squamous cells driven by HPV infection, showing maturation abnormalities and/or viral cytopathic changes that do not extend beyond the basement membrane [3]. CINs are graded as CIN1, CIN2, and CIN3, according to the extent of abnormal proliferation in the atypical basal/parabasal-like cells and mitotic activity [3]; in CIN1, atypical proliferation and mitosis occur up to the lower third of the epithelium along with koilocytotic atypia with clearly retained features of maturation. CIN2 shows basal/parabasal morphology and mitotic activity extending into the lower two-thirds of the epithelium, but with maturation in the uppermost cell layers. CIN3 demonstrates full-thickness basal/parabasal-type atypia and mitotic activity without maturation in the top-most epithelial layers. Recently, due to the improved reproducibility and enhanced biological relevance, a two-tier terminology of low-grade squamous intraepithelial lesion (LSIL), which includes CIN1, and high-grade squamous intraepithelial lesion (HSIL), which may be subdivided into CIN2 and CIN3 is preferred in premalignant lesions of the cervix [2]. However, the CIN classification still has clinical importance. In the natural clinical course, LSIL has a low potential for progression and a high potential for regression, which it has been conservatively managed [4,5,6]. In contrast, HSIL was actively treated for cure due to a higher potential for progression and a lower potential for regression and the treatment was standardized irrespective of CIN2 and CIN3 [5]. In recent studies, the higher regression rates of CIN2 unlike CIN3 have led to the adoption of alternative conservative management strategies in women who wish to preserve fertility [6]. Consequentially, the updated guidelines by the American Society of Colposcopy and Cervical Pathology (ASCCP) in 2019 strongly recommended to qualify a histologic HSIL result by CIN2 or CIN3 for epidemiologic and clinical management purposes [6]. However, pathologists often encounter difficulties in accurately diagnosing and grading CIN [7]. The effects of inflammation, repair, pregnancy, and atrophy, as well as the inherent difficulty in distinguishing lesions with a morphologic spectrum, complicate it and may lead to substantial inter-observer and intra-observer variability [7,8,9]. The time pressure, workload, and limited experience of the pathologist may be other hindrances. With the increase in cervix specimens due to population growth, increased prevalence of cancers, and longer life spans, these obstacles will likely worsen in the future. In addition, due to the limited well-trained pathology workforce, the quality of pathology services is uneven nationwide and worldwide [10]. The use of automatic histology image classification can alleviate the scarcity in professional resources and heavy workloads.

With the advancement of artificial intelligence (AI), machine learning techniques can be used as a major ancillary tool for diagnosing tumors in various organs based on the histological images. However, most recent studies have applied techniques for the detection and classification of invasive cancers [11,12,13,14,15,16] rather than intraepithelial or premalignant lesions. With regard to cervical lesions, some studies have been devoted to the creation of computer-assisted reading systems for assessing cervical cytology specimens [17,18] and only a limited number of studies have focused on examining CINs [19,20,21,22,23,24,25]. In this study, we aimed to develop and assess an optimal convolutional neural network (CNN) model for classification of CINs.

## 2. Materials and Methods

### 2.1. Data Collection

Female patients who were scheduled for colposcopic biopsy or conization due to suspicion of CIN at Kangnam Sacred Heart Hospital between 2015 and 2017 were retrospectively enrolled. This study was approved by the Institutional Review Board (IRB) of Kangnam Sacred Heart Hospital (IRB no. HKS 2018-03-013) and performed in accordance with the Declaration of Helsinki. One experienced pathologist (J-W.K.) reviewed the histological slides of tissue sections of the involved patients that were stained with hematoxylin and eosin (H&E) and p16 antibody (Roche E6H4^TM^, catalog #725-4713) and obtained digital microscopic photographs of representative lesions at an objective magnification of 20× using a microscope (Olympus BX51; Melville, NY, USA) equipped with a digital camera (Olympus DP2). Photographic images were acquired in JPEG format with a resolution of 2560 × 1920 or 1280 × 960 pixels. Unsuitable blurred or defocused images were excluded from this study. Three experienced pathologists (J-W.K., M.H., and G-Y. K.) were independently re-reviewed the digitalized H&E images along with the corresponding p16 immunohistochemical images. Blinded to the results of other pathologists, according to the 2019 World Health Organization (WHO) classification [3] and the 2012 Lower Anogenital Squamous Terminology (LAST) standardization project [2] the three pathologists classified the images into four classes: CIN3, CIN2, CIN1, and non-neoplasm. On H&E images, CIN1 was defined as a proliferation of basal/parabasal-like cells and mitosis (not atypical) restricted to the lower third of the epithelium along with koilocytotic atypia within the middle and surface cells. CIN2 was characterized by atypical basaloid cells and mitotic activity extending into the upper half to upper two-thirds of the epithelium, but with retained koilocytotic changes or maturation on the surface. CIN3 was defined as full-thickness basal/parabasal-type atypia and mitotic activity without maturation in the top-most epithelial layers. To aid in the distinction of CIN2/CIN3 from mimickers of precancer and CIN1, p16 immunohistochemistry (IHC) was adjunctively used [2,3]. Out of 1305 H&E images, 199 (15.2%) with any discrepancy were excluded and only images categorized in the same class by the all three pathologists were involved in this study. Ultimately, 1106 microscopic images from 588 patients were included: 266, CIN3; 231, CIN2; 266, CIN1; and 343, non-neoplasms (Table 1).

### 2.2. Dataset Construction

From the whole dataset, the test dataset was randomly split three times with a ratio of 10% to evaluate the performance of the trained CNN models. Random train/test set splitting was performed for each class using the patient ID as the key to avoid the simultaneous involvement of the same class image of one patient in both the training and test datasets. For each three splitting, the training set consisted of 90% of the whole dataset and was divided into the training dataset proper and the tuning (or validation) dataset, with a ratio of 80%:10%. Therefore, each CNN model was trained three times independently using three different split folders.

### 2.3. Dataset Preprocessing

All images were resized to 640 × 480 pixels reducing the resolution of the whole images and were normalized for each RGB color channel based on the mean and standard deviation values of the images in the ImageNet dataset. Data augmentation and histogram equalization were not performed as these methods did not improve the model performance in our pilot studies.

### 2.4. Deep Learning Model Training

Two CNN architectures were adopted: DenseNet-161 and EfficientNet-B7. The details of the CNN models are described in previous studies [26,27]. Briefly, DenseNet-161 is characterized by a dense block that uses the feature maps of the previous layers as the input of the current layer [26]. EfficientNet is characterized by an MBconv block that balances the width and depth of the CNN via reinforcement learning [27]. These models were pre-trained using the ImageNet Large Scale Visual Recognition Challenge dataset and fine-tuned using the training dataset of this study.

In the first experiment, the CNN models were trained to perform four-class classification that classified images into CIN3, CIN2, CIN1, and non-neoplastic lesions. Then, the CIN2 and CIN3 groups of the whole dataset were merged into one group, representing HSIL. In the second experiment, the training/test set splitting was re-performed; the CNN models were trained to perform three-class classification and classified the images into CIN2–3 and CIN1, which represent LSILs, and non-neoplasms.

The model was trained using the PyTorch platform with categorical cross-entropy as the loss function. The Adam optimizer was adopted with a β1 value of 0.9 and a β2 value of 0.999. The learning rate was 1 × 10^−4^, and the batch sizes were 15 and 5 for DenseNet-161 and EfficientNet-B7, respectively. The number of training epochs was set to 100, and the model with the minimum validation loss was chosen. The hardware platform was equipped with NVIDIA GeForce GTX 1080ti 6-way graphics processing units, dual Xeon central processing units, 128 GB RAM, and a customized water-cooling system.

Saliency maps were produced to identify the regions of interest with a gradient-weighted class activation mapping (Grad-CAM) [28]. Grad-CAM method can highlight a class-specific local features in the image using gradient information. Overall, there are three steps to generate a class-specific saliency map. First, it computes the gradient of the logit for predicted class with respect to the last CNN layer which not only learns high-level abstract features but also retains spatial information. Then, the gradients are global-average-pooled to estimate the importance of feature maps in the last layer. Lastly, along with the importance weights, feature maps are averaged. In order to highlight only features that actually increase the value of class logit, ReLU function is applied to the averaged feature map [28]. In this study, an implementation of Grad-CAM for PyTorch-based models was used (available at: https://github.com/jacobgil/pytorch-grad-cam; accessed on 10 June 2021).

### 2.5. Human Performance Evaluation

For the first test dataset, two other experienced human pathologists, who were blinded to the true labels, independently classified the images, and the performances were evaluated. Human performances were compared with those of the CNN models.

### 2.6. Main Outcome Measures and Statistical Methods

The primary outcome was the model performance for four-class classification, while the secondary outcome was model performance for three-class classification. The performance of the CNN model was evaluated using three different test datasets, and the performance was estimated using means and 95% confidence intervals (CIs). The performance was evaluated using the diagnostic accuracy and the area under the receiver operating characteristic (ROC) curve (AUC). For each class, per-class sensitivity, specificity, positive predictive value, and negative predictive value were also evaluated. Continuous or categorical variables are expressed as means or percentages with 95% CIs. Statistical significance was set at *p* < 0.05.

## 3. Results

A total of 1106 images from 588 patients were included in this study. The patients’ mean age was 43.0 ± 12.4 years (range: 16–84 years). The patients’ data are presented in Table 1. Non-neoplastic lesions comprised the majority class (343 cases from 250 patients, 31.0%) in the whole dataset, while CIN2 was the least common type (231 cases, 20.9%). The test dataset for the human performance evaluation comprised 117 images from 68 patients.

### 3.1. Four-Class Classification Performance of Deep Learning Models and Human Pathologists

**Figure 1:**
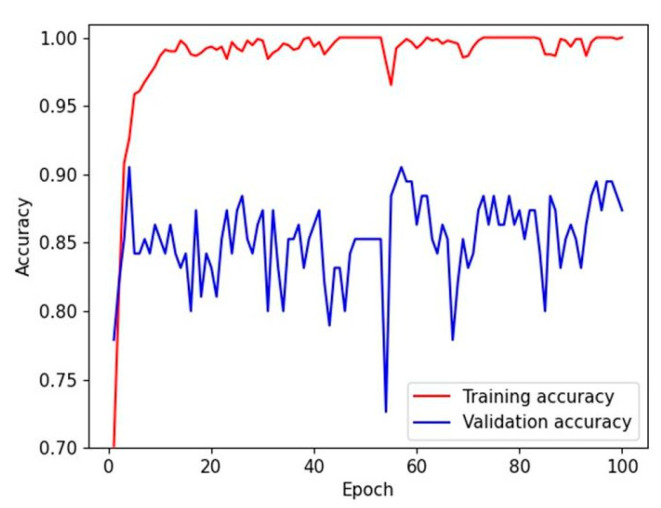
A training curve for training and validation accuracies. The validation accuracy reached a plateau within 20 epochs during model training.

**Figure 2:**
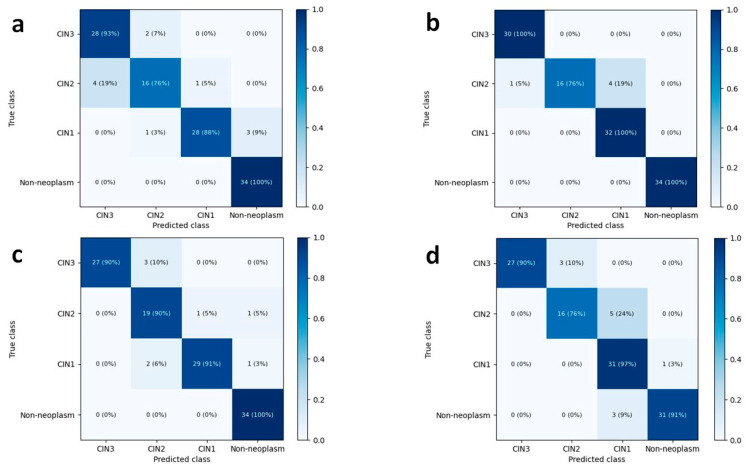
Heatmaps for confusion matrix of the best-performing CNN models and human pathologists in the four-class classification. There were three false-negative cases in the best-performing DenseNet-161 (**a**) model, there was no false-negative or false-positive case with the best-performing EfficientNet-B7 (**b**). Pathologist 1 (**c**) classified CIN2 with higher sensitivity than pathologist 2 (**d**).

The mean accuracies for the four-class classification (CIN3, CIN2, CIN1, and non-neoplasm) in the test dataset were 88.5% (95% CI, 86.3–90.6%) by DenseNet-161 and 89.5% (83.3–95.7%) by EfficientNet-B7, respectively (Table A1). The validation accuracy reached a plateau within 20 epochs during the model training, as shown in Figure 1. The overall accuracies for the four-class classification of human pathologists were 93.2% and 89.7%, respectively. The heatmaps for the confusion matrix of the best-performing models for the test dataset and human pathologists are presented in Figure 2.

**Figure 3:**
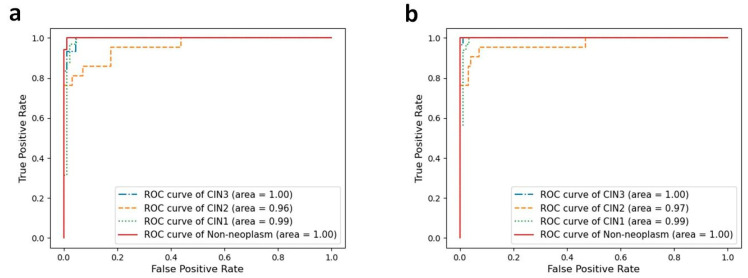
Per-class ROC curves for four-class classification the best-performing CNN models. For DenseNet-161 (**a**) and EfficientNet-B7 (**b**) with best performance, AUC was higher in discriminating non-neoplasm and CIN3 rather than in classifying CIN2 and CIN1.

The per-class performances of the deep learning models are presented in Table 2 and Figure 3 depicts the per-class ROC curves for the best-performing CNN models. For both CNN architectures, the mean AUC was highest in discriminating non-neoplastic lesions (0.996 for DenseNet-161 and 0.996 for EfficientNet-B7). For both CNN architectures, the mean AUC was lowest in discriminating CIN2 lesions, but the individual AUCs remained high (0.947 for DenseNet-161 and 0.956 for EfficientNet-B7, respectively). In determining CIN3 lesions, EfficientNet-B7 showed a mean sensitivity of 97.5% (95.4–99.5%) and a mean specificity of 96.3% (94.1–98.6%). For the CIN1 lesions, EfficientNet-B7 presented a mean sensitivity of 85.2% (73.3–97.1%) and a mean specificity of 96.3% (95.1–97.6%).

### 3.2. Histologic Review of Misclassified Cases in Four-Class Classification Using Best-Performing CNN Models

**Figure A1:**
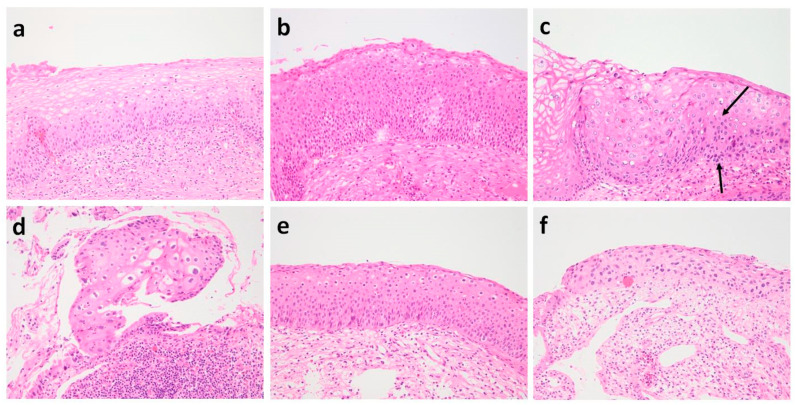
Histology of misclassified cases by CNN models. A case with scarce koilocytotic cells but basal atypia was false-negative (**a**). CIN3 showing basal/parabasal-type atypia throughout most of the epithelium but not all was downgraded to CIN2 (**b**). CIN2 (**c**) downgraded as CIN1 showed koilocytotic changes in the upper half and maturation in upper most layers but had atypia focally extending to the lower half of the epithelium (black arrow). In CIN1 upgraded as CIN2, the epithelium was disoriented (**d**). CIN2 with koilocytosis (**e**) and atrophic CIN2 (**f**) were upgraded as CIN3.

No false-positive cases were included in the best-performing CNN models, both DenseNet-161 and EfficientNet-B7 (Figure 2). False-negative cases were not observed by EfficientNet-B7; among 117 test cases, three CIN1s (2.6%) were classified as false-negative cases by DenseNet-161. After a histological review, it appeared that the scarcity of characteristic koilocytotic cells might have contributed to the misclassification (Figure A1a).

Eight (9.2%) out of 87 CIN cases were unsuccessfully graded by DenseNet-161; one CIN3 (1.1%) and two CIN2 (2.3%) cases were downgraded as CIN1 and CIN2, respectively, while one CIN1 (1.1%) and four CIN2 cases (4.6%) were upgraded as CIN2 and CIN3, respectively (Figure 2a). EfficientNet-B7 misgraded the five CIN2 cases (5.7%): four cases were classified as CIN1, while one case was classified as CIN3 (Figure 2b). None of the CIN1 cases were classified as CIN3, and none of the CIN3 cases were classified as CIN1. On histological review, histology of CIN3 cases downgraded as CIN2 was not sufficient to be classified as carcinoma in situ. The cases showed basal/parabasal-type atypia throughout the full-thickness of the epithelium (Figure A1b). CIN2 cases downgraded as CIN1 had atypia extending to the lower half of the epithelium with koilocytotic changes in the upper half and maturation in the uppermost layers (Figure A1c). The CIN1 case upgraded as CIN2 showed disoriented epithelium (Figure A1d). One of the CIN2 cases upgraded as CIN3 showed atrophy (Figure A1f).

### 3.3. Three-Class Classification Performance of Deep Learning Models and Human Pathologists

**Figure 4:**
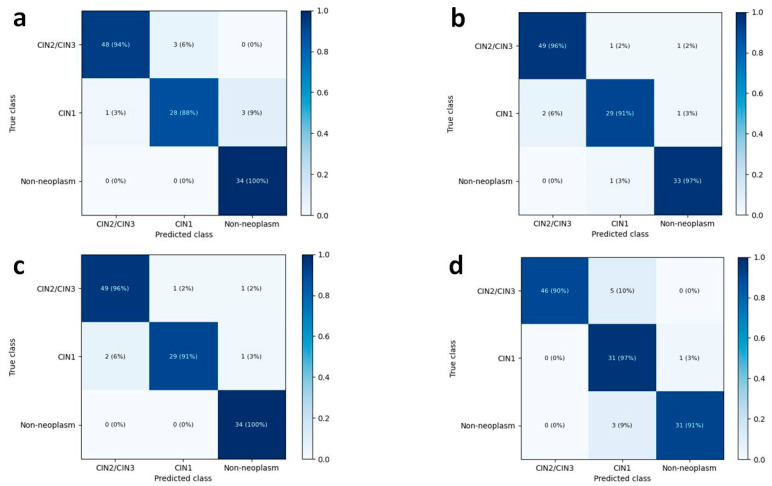
Heatmaps for confusion matrix of the best-performing CNN models and human pathologists in the three-class classification. The overall accuracies increased up to 94.0% by DenseNet-161 (**a**) and 94.9% by EfficientNet-B7 (**b**), similar to those of human pathologists 1 and 2, 95.7% (**c**) and 92.3% (**d**), respectively.

In the three-class classification discriminating the images into CIN2–3, CIN1, and non-neoplasm, the mean accuracies in the test dataset increased up to 91.4% (95% CI, 88.8–94.0%) by DenseNet-161 and 92.6% (95% CI, 90.4–94.9%) by EfficientNet-B7. The overall accuracies for the three-class classification of human pathologists were 95.7% and 92.3%, respectively. Figure 4 shows the heatmaps of the confusion matrix of the best-performing models for the test dataset and human pathologists.

The per-class performances of the deep learning models in the three-class classification are listed in Table 3. The mean AUCs for non-neoplastic lesions were 0.996 (95% CI, 0.992–0.999) for DenseNet-161 and 0.993 (95% CI, 0.985–1.000) for EfficientNet-B7. The mean AUCs for CIN2–3 and CIN1 were 0.981 and 0.974 for DenseNet-161 and 0.982 and 0.979 for EfficientNet-B7. In terms of determining CIN2–3 lesions, EfficientNet-B7 showed a mean sensitivity of 94.8% (92.7–96.7%) and a mean specificity of 93.4% (90.1–96.8%).

### 3.4. Analysis of Grad-CAM Images by CNN Model

**Figure 5:**
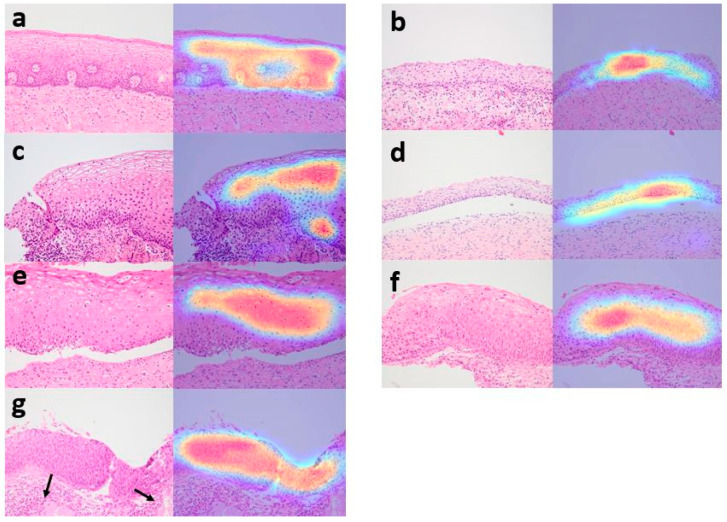
Grad-CAM images by EfficientNet-B7. Normal squamous epithelium was highlighted in Grad-CAM images (**a**–**d**). Images from cervix interpreted as non-neoplasm by the EfficientNet-B7 include exocervix (**a**), metaplastic muco-sa from transformation zone (**b**), cervicitis and erosion (**c**) and atrophic mucosa (**d**). In CIN1, layers with koilocytotic cells were mainly highlighted (**e**). The highlighted areas extended to the upper two-third of the epithelium in CIN2 (**f**) and full-thickness of the epithelium in CIN3 (**g**). Normal endocervical glands ((**g**), black arrows) were not highlighted.

Figure 5 shows the representative Grad-CAM images of non-neoplasms, CIN1, CIN2, and CIN3. Grad-CAM images were reviewed by a pathologist, and the region of interest of the deep learning model agreed with that of humans. The CNN model successfully detected squamous epithelium and recognized images from the transformation zone and exocervix, atrophic cervix, and cervicitis with erosion as non-neoplasms. In Grad-CAM images, CIN1, CIN2, and CIN3 characterized by presence of koilocytotic cells or hyperchromatic atypical cells with a high nuclear/cytoplasmic ratio and increased mitotic activity were depicted as highlighted areas. According to the distribution of abnormal cells, different layers of squamous epithelium were highlighted. 

## 4. Discussion

In recent years, AI has been used in the field of pathologic image diagnosis, and many studies have shown promising results in detecting and diagnosing cancers in a variety of organs, including the stomach [29], colon [15], breast [30], prostate [16], head and neck [13], brain [14], and lungs [31]. As for cervical cancer, with the advancement in the management of preinvasive lesions, the increasing diagnostic workload of cervical biopsy calls for the development of high-performance algorithms with high sensitivity and specificity. Practically, many pathologists experienced more difficulty and burden in accurately classifying preinvasive cervical lesions than in distinguishing between invasive and non-neoplastic condition [7]. Therefore, we focused on developing an optimized CNN system for CIN grading. 

For the classification of premalignant lesions of the cervix, LSIL and HSIL have been the preferred terminology in both tissue and cytology specimens due to the improved reproducibility and biological relevance of the two-tier system [2]. A recent systemic review and meta-analysis of studies from 1973 to 2016 [32] indicated that among CIN2 managed conservatively, 50% regressed, 32% sustained, and 18% progressed to CIN3. The regression rate of CIN2 was higher (60%) in particular in women younger than 30 years and observation has been acceptable for CIN2 in the women [6,32]. Consequently, the subdivision of HSIL into CIN2 and CIN3 is essential for making treatment decision for young women. Hence, we investigated the four-class classification (CIN3, CIN2, CIN1, and non-neoplasm) performance as well as the three-class classification (CIN2-3, CIN1, and non-neoplasm) performance.

Two CNN architectures, DenseNet-161 and EfficientNet-B7, were adopted in our study. EfficientNet-B7 is a recently developed heavy model and a state-of-the-art architecture; it showed better performance than DenseNet-161 [27]. However, DenseNet-161 also showed excellent performance and presented good cost-effectiveness [26]. In the four-class classification, the mean accuracies for DenseNet161 and EfficientNet-B were 88.5% and 89.5%, respectively, and the performance was similar to that of human pathologists (93.2% and 89.7%, respectively). The mean AUC values of both CNN models were considerably high in all four classes (Table 1). Furthermore, the mean accuracies of both models for three-class classification were increased to 91.4% and 92.6% by DenseNet-161 and EfficientNet-B7, respectively, which are almost the same levels as those of human pathologists (95.7% and 92.3%, respectively).

Compared to the four-class classification, the three-class classification had imbalanced classes due to merging CIN2 and CIN3. The numbers of CIN2/3, CIN1, and non-neoplasm were 497, 266, and 343 and the CIN2/3:CIN1 ratio was 1.87. Although the ratio of CIN2/3 to CIN1 (or non-neoplasm) increased, the model learned the features of CIN1 and CIN0 and showed high accuracy for the minority classes. In fact, the per-class AUCs for CIN1 and non-neoplasm in EfficientNet reached 0.993 and 0.979, respectively. Consequently, the mean accuracies of the CNN models for three-class classification were increased than those for four-class classification. In other similar studies, the imbalanced datasets were used without a balancing strategy [33,34]. In the light of previous other studies and our experimental results, we concluded that the imbalance was not a critical issue in our study. However, in both four-class classification and three-class classification by EfficientNet-B7, the mean F1 scores of the minority classes, CIN2 and CIN1, respectively, were 79.1 and 86.8, which were lower than those of the majority classes. Balancing datasets and building large datasets might increase the F1 scores in the future research.

Through repeated validation and tests of two CNN models, we determined the optimal image preprocessing conditions (640 × 480 pixels size and normalization of each RGB color channel in the ImageNet dataset) in which the CNN models can achieve better performance. The most suitable resolution for deep learning of the histopathological images is not known yet. Since the preprocessing method for high-resolution and large-scale histopathological images causing memory limitation can induce important information loss, it must be handled carefully. Various methods to overcome loss of information have been reported recently [35]. In the early stage of this study, we tried three different image resolutions for our four-class classification, and observed that the resolution of 640 × 480 was not inferior to 1280 × 960 and 800 × 600 (data not shown). After the experiment, we decided to continue our experiments with 640 × 480 image resolution considering the training and evaluation speed. Since at 640 × 480 image resolution, the deep learning models showed a similar level to human performance in four-class classification, we can draw conclusions that the deep learning models perform effectively even after resizing the CIN images and the resolution of 640 × 480 is appropriate for this study. In addition, we found that data augmentation and histogram equalization did not improve the model performance. To overcome a small number of training datasets in CNN, image augmentation has been frequently used. In our pilot experiments applied horizontal-flip and contrast limited adaptive histogram equalization (data not shown), the model performance did not significantly change depending on the augmentation methods. Thus, we concluded that the augmentation methods are not likely to boost the model performance in this study.

Approximately 90% of CIN1 regress without treatment, and less than 1% progress to invasive cancer, whereas the risk of progression of untreated CIN2 and CIN3 to cancer is estimated to be 0.5–1% per year [4]. Notably, CIN3 is a direct precursor of invasive cervical cancer, and active treatment is recommended. By contrast, observation is the preferred approach for CIN1. Therefore, it is much more critical to quickly determine CIN1 or CIN3 than CIN2 or CIN3. Despite some misclassification of CIN2, EfficientNet-B7 perfectly discriminated CIN1 from CIN3 based on the four-class classification (Figure 2), and the results demonstrated its clinical applicability.

We observed that the CNN models have a weakness in classifying CIN2 (75.2% and 73.0% sensitivities for DenseNet-161 and EfficientNet-B7, respectively). Considering that it is often challenging for pathologists to distinguish CIN2 from CIN1 and CIN3 and inter-observer, agreement is notoriously poor at this interface, even among experts [4], the performance of these CNN models is almost similar to that of human pathologists even in this respect. Moreover, the difficulty in classifying CIN2 can be attributed to its inherent nature, which is intermediate in the morphological spectrum of CIN. Due to the ambiguity of CIN2 diagnosis based on the H&E morphology, the LAST Project suggested that the addition of p16 immunohistochemical stain significantly improves the reliability of CIN2 diagnosis and advised the use of p16 staining to confirm the presence of a high-grade lesion when CIN2 is diagnosed based on H&E slide [2]. In future studies, analyzing H&E images along with the images of p16 immunohistochemical staining would be helpful to increase diagnostic accuracy of CNN models.

For determining the CIN1 lesions, the mean sensitivities were 82.1% and 85.2% by DenseNet-161 and EfficientNet-B7, respectively, which were lower than those of CIN3 and non-neoplasms. On histologic review, the scarcity of characteristic koilocytotic cells in CIN1, severe inflammation, and metaplastic changes might have contributed to the inaccuracy of CNN classification. For more precise detection of koilocytotic cells, the CNN model needs to be improved. To reduce the false-positive rate, more variable non-neoplastic lesions, such as chronic cervicitis, metaplastic mucosa, and atrophy, should be included in the study set, and a repeat validation would be helpful.

Automated screening machines have been developed for analyzing cervical cytology smears, and a few FDA-approved automated primary screening device are available [17]. However, it is more difficult to develop an automated tool for cervical tissue histology due to the complexity of the patterns observed and the structural associations between different tissue components. Keenan et al. [21] developed a machine vision system for histological grading of CIN using the KS400 macro programming language. It was a scoring system that analyzes geometric data, and 62.7% of the CIN cases with captured images were correctly classified [21]. Several previous studies have used multiclass support vector machines and gray-level co-occurrence matrices to analyze whole slide images (WSIs) or selected images [22,25,36]. Despite some promising results, the small data size of less than 100 cases with insufficiently validated or curated images and the extremely complicated methodology limited the applicability of the study results. Huang et al. [23] proposed a method based on the least absolute shrinkage and selection operator and ensemble learning support vector machine. They showed that the accuracy of normal-cancer classification was high (99.64%), but the accuracy of the LSIL-HSIL classification was 76.34%. A recent study that classified cervical tissue pathological images based on fusing deep convolution features has been published [37]. The researchers analyzed the dataset comprising small-sized images cropped from 468 WSIs, including those of normal tissues, LSIL, HSIL, and cancer. Resnet50v2 and DenseNet121(C5) showed excellent performance, with an average classification accuracy of 95.33%.

Pathologic classification is an image-based method, and CNN is an optimized AI tool for image learning. Our study showed that CNN is a robust instrument for pathologic classification, but some things must be considered. For CNN to be developed and to work properly, collecting a large amount of accurate data is of utmost importance. Since CNN produces results very faithfully in the learned input, the quality of the CNN output absolutely depends on the quality of the input data. In order to develop a clinically relevant CNN model for pathologic diagnosis, a superb dataset from expert pathologists must be constructed. Recently, Meng et al. provided a public cervical histopathology dataset for computer-aided diagnosis, called MTCHI [24]. Pathologic diagnosis is sometimes equivocal and might be challenging to perform in some lesions in the gray zone or lesions with reactive changes. Therefore, pathologists should continue to improve and make an objective pathological diagnosis. In addition, high-quality H&E slide images is the need for using AI to perform a pathologic diagnosis. Although staining and mounting are automated, preparing pathology slides, sectioning, and embedding are still manually performed. Artifacts in the production process, such as tissue overlapping, tangential embedding, and poor sectioning, hinder the acquisition of focused images and cause AI to make diagnostic errors.

We aimed to develop an artificial technique for classifying CIN from the WSI of cervical biopsy, but some practical difficulties were observed. In the WSIs of tissues, grading of intraepithelial neoplasia or dysplasia is much more complicated than finding lesions or cancer. Since CIN is a morphological spectrum, cervical biopsy specimens show large differences in disease degrees and mix of lesions. This makes it difficult for pathologists to precisely annotate according to the CIN grade in small biopsies. Compared with other tissues such as the breast, colon, and stomach, the specimen used for cervical biopsy are tissue strips or appear irregular in shape and often include a small amount of epithelium. Moreover, it is easily embedded in a disoriented or tangential manner. These were obstacles in making a standardized dataset using WSIs suitable for training and validation of the CNN model. In this study, we built a reliable dataset of CIN provided by three qualified pathologists and analyzed the CNN performance prior to its application in WSI. For enabling the future broad application of AI-based pathology in cervical biopsy, it is essential to build a large-scale multicenter dataset with a standardized protocol. It is another limitation of our study that there was still a gap between the training and validation accuracies, although we tried several strategies for image normalization, data augmentation, and loss function optimization. Novel approaches for these issues might improve the final model performances in the future. 

In conclusion, we built a reliable dataset for CIN classification and showed that EfficientNet-B7 and DenseNet-161 provided a promising performance in classifying cervical lesions on digital histology images. In terms of accuracy, EfficientNet-B7 had a functional advantage over DenseNet-161. Grad-CAM images used in the CNN models located the areas where CIN lesions can be found. Moreover, we realized that the accurate identification and classification of CIN by CNN relies entirely on the standardized diagnosis of pathologists, and the professional knowledge and analytical experience of pathologists are the cornerstone of technical advancement. An exquisite AI tool trained using a well-established and standardized dataset would be helpful in improving the pathology services worldwide.

## Figures and Tables

**Table 1:** Data composition for the first splitting of the training and test datasets.

	Whole Dataset		Training Set		Test Set	
	Image N	Patient N	Image N	Patient N	Image N	Patient N
Overall	1106	588	989	542	117	68
CIN 3	266	183	236	165	30	19
CIN 2	231	108	210	97	21	11
CIN 1	266	143	234	129	32	14
Non-neoplasm	343	250	309	225	34	25

N, numbers; CIN, cervical intraepithelial neoplasia.

**Table 2:** Per-class performances of the deep learning models in the four-class classification.

Model/Class	Sensitivity (%)	Specificity (%)	PPV (%)	NPV (%)	F1 Score	AUC (95% CI)
DenseNet-161						
CIN3	95.3 (93.7–96.8)	94.4 (93.1–95.6)	85.0 (82.3–87.7)	98.3 (97.8–98.9)	89.8 (88.8–90.8)	0.989 (0.982–0.996)
CIN2	75.2 (67.7–82.8)	94.1 (91.8–96.4)	76.1 (62.3–89.9)	93.8 (92.5–95.0)	75.5 (64.9–86.1)	0.947 (0.932–0.963)
CIN1	82.1 (77.8–86.5)	98.3 (97.4–99.2)	94.2 (92.2–96.2)	94.5 (92.6–96.4)	87.7 (84.5–91.0)	0.979 (0.968–0.990)
Non-neoplasm	95.6 (90.9–100.0)	98.0 (96.3–99.7)	95.0 (91.0–99.0)	98.3 (96.6–100.0)	95.2 (92.0–98.4)	0.996 (0.991–1.000)
EfficientNet-B7						
CIN3	97.5 (95.4–99.5)	96.3 (94.1–98.6)	90.0 (84.2–95.8)	99.1 (98.4–99.8)	93.6 (89.6–97.5)	0.990 (0.981–0.999)
CIN2	73.0 (62.2–83.9)	96.7 (93.7–99.7)	86.8 (75.2–98.4)	93.6 (92.3–94.8)	79.1 (69.1–89.1)	0.956 (0.946–0.967)
CIN1	85.2 (73.3–97.1)	96.3 (95.1–97.6)	88.5 (88.2–88.8)	95.5 (91.3–99.8)	86.5 (80.4–92.6)	0.971 (0.950–0.993)
Non-neoplasm	95.6 (90.9–100.0)	96.3 (92.2–100.0)	92.3 (84.8–99.8)	98.3 (96.6–100.0)	93.8 (88.8–98.8)	0.996 (0.992–0.999)

PPV, positive predictive value; NPV, negative predictive value; AUC, area under the receiver operating characteristic curve; CI, confidence interval.

**Table 3:** Per-class performances of the deep learning models in the three-class classification.

Model/Class	Sensitivity (%)	Specificity (%)	PPV (%)	NPV (%)	F1 Score	AUC (95% CI)
DenseNet-161						
CIN2-3	92.0 (86.9–97.1)	92.4 (85.3–99.6)	92.5 (87.0–98.0)	93.4 (90.2–96.7)	92.1 (88.9–95.3)	0.981 (0.973–0.989)
CIN1	80.9 (70.9–90.8)	96.0 (94.2–97.7)	87.0 (84.0–89.9)	94.5 (93.3–95.6)	83.5 (77.6–89.4)	0.974 (0.968–0.980)
Non-neoplasm	97.8 (94.2–100.0)	97.5 (95.6–99.5)	94.4 (90.0–98.9)	99.1 (97.6–100.0)	95.9 (95.5–96.4)	0.996 (0.992–0.999)
EfficientNet-B7						
CIN2-3	94.8 (92.8–96.7)	93.4 (90.1–96.8)	92.9 (90.3–95.6)	95.1 (92.3–97.9)	93.8 (91.7–96.0)	0.982 (0.971–0.993)
CIN1	86.1 (82.4–89.7)	96.4 (95.2–97.5)	87.6 (81.2–94.0)	95.6 (94.3–96.9)	86.8 (82.1–91.4)	0.979 (0.972–0.985)
Non-neoplasm	94.7 (92.8–96.6)	98.4 (97.0–99.7)	96.0 (92.8–99.2)	97.8 (97.1–98.6)	95.3 (94.0–96.6)	0.993 (0.985–1.000)

PPV, positive predictive value; NPV, negative predictive value; AUC, area under the receiver operating characteristic curve; CI, confidence interval.

## Data Availability

The data that support the findings of this study are available from the corresponding author up-on reasonable request.

## Appendix A

**Table A1:** Accuracies of deep learning models.

	Four-Class Classification		Three-Class Classification	
	DenseNet-161	EfficientNet-b7	DenseNet-161	EfficientNet-b7
Mean accuracy	0.885	0.895	0.914	0.926
95% CI	0.863–0.906	0.833–0.957	0.888–0.940	0.904–949
Test 1	0.906	0.957	0.940	0.949
Test 2	0.873	0.853	0.901	0.919
Test 3	0.875	0.875	0.901	0.911

CI, confidence interval; CI, confidence interval.
